# 14154 Facilitating Community/Campus Research Teams and Projects: Community Health Small Grants Program

**DOI:** 10.1017/cts.2021.741

**Published:** 2021-03-30

**Authors:** Sharon A. Croisant, John Prochaska, Chantele Singleton, Krista Bohn, Lance Hallberg, Lori Wiseman, Eleanor Hanley, Lesley Sommer

**Affiliations:** 1 The University of Texas Medical Branch; 2 Alcohol and Drug Abuse Women’s Center and REACH Coalition; 3 Galveston Association of Realtors and REACH Coalition

## Abstract

ABSTRACT IMPACT: The UTMB Institute for Translational Sciences (ITS) seeks to advance the field of community engagement and facilitate competency in community-engaged and community-based participatory research as a means of expanding team science to integrate community involvement and to assist investigators in building relationships that enable them to contribute to community initiatives. OBJECTIVES/GOALS: The UTMB ITS recently implemented a new Community Health Small Grants program to promote and enhance community-campus partnerships. Our goal is to better translate science from discovery to clinical practice and public health through community-engaged research, education, and dissemination. METHODS/STUDY POPULATION: Applications were solicited from community and academic research partners. Community partners may include existing collaborative groups, community health centers, health departments, nonprofits, schools, social services agencies, practice-based research networks, or Community Advisory Boards. Academic partners may include faculty and/or students. The PI may be a community or academic partner. While this Grants Program will transition to the ITS Pilot Project Program, it will utilize a separate review process and scoring rubric focused on immediate and future community benefit, project feasibility, organizational fit, and other factors unique to community-based partnership projects. RESULTS/ANTICIPATED RESULTS: We received an enthusiastic response to our RFA, based upon a long-standing program of a sister CTSA hub. Proposals received include target populations representative of our most vulnerable’‘ children, the elderly, those lacking access to health care, and those for whom language is a barrier. One addresses the Institution’s and the CEC’s need to conduct community needs assessments to enable the implementation of evidence-based programs driven by data and metrics identified and developed by our communities. Each awarded proposal demonstrates a significant and sometimes critical need for the project. Partnerships are anticipated to have significant impacts on the community and its population. DISCUSSION/SIGNIFICANCE OF FINDINGS: We generate, test, and disseminate team science, education and best practices through stakeholder involvement. Our Community Health Small Grants program aims to involve community in our scientific teams and to involve academics in community-derived projects as well as foster relationships and trust.

